# Nurse Disciplinary Leadership in Higher Education: A Scoping Review

**DOI:** 10.1155/jonm/2161553

**Published:** 2025-04-20

**Authors:** Melissa Slattery, Carol Grech, Rachael Vernon, Jennifer Fereday

**Affiliations:** ^1^Clinical & Health Sciences Academic Unit, University of South Australia, Adelaide, Australia; ^2^Rosemary Bryant AO Research Centre, University of South Australia, Adelaide, Australia; ^3^Mental Health and Suicide Prevention Research and Education Concentration, University of South Australia, Adelaide, Australia

**Keywords:** academic, core attributes, higher education, leadership and management, nursing

## Abstract

**Background:** Leadership of nurse education and research has become increasingly challenging over the last decade and remains a global issue affecting nurse managers working in the higher education (HE) sector. Nursing faculties have not been immune from the effects of funding constraints, policy reforms and organisational restructures.

**Aim:** This scoping review explored what is currently known about the qualities, behaviours and characteristics (core attributes) of nurse disciplinary leads that manage nursing faculties in HE.

**Method:** Five research questions were identified, and a scoping review method was used to map a wide-ranging set of literature and concepts. Databases were searched using a prospective protocol tool, and an extraction table was created to facilitate content analysis.

**Findings:** Ten articles that addressed one or more of the research questions were identified. While the literature described a range of core attributes required to lead nursing faculties in HE, clarity and definition of terms used were lacking, and articles were primarily opinion-based and nonevidential.

**Discussion:** Whilst key leadership core attributes may be transferable, nurse disciplinary leads require specific knowledge of the HE sector to operate effectively in this environment. Mentorship was reported to have a highly positive impact on academic nurse leadership skill development.

**Conclusion:** Further research is needed to identify opportunities to support the growth and readiness of future generations of nurse disciplinary leads and challenge the current deterrents inhibiting nurse academic career pathways in the Australian and New Zealand HE sectors.

## 1. Introduction

Nursing is a well-established academic discipline in the higher education (HE) sector, creating knowledge through research inquiry and producing graduates for employment in health via professionally accredited and nonaccredited programs. Although nursing is primarily recognised as a clinical practice-based profession, registered nurses (RNs) seeking career opportunities in education and/or research have been attracted to academic positions in HE. RNs moving into HE roles most often do so after significant years of clinical experience. This factor along with the requirement to gain postgraduate qualifications for career advancement in HE means that nurse academics are often an older demographic group with limited time to attain senior leadership roles that can positively shape and lead nurse education and research into the future [1]. Early identification of individuals with leadership capabilities will help guide succession planning for nurse disciplinary lead roles despite the challenges faced by a reduction in nursing professionals entering the education sector in contrast with a predicted increase in demand for tertiary education [1, 2].

As multiple leadership position titles are used across the HE sector, for the purpose of this study we used the term ‘nurse disciplinary lead' to refer to the most senior faculty manager appointment (e.g. Head of Discipline for Nursing, Head of School of Nursing, or Dean of Nursing). In the Australian and New Zealand HE sectors, this is usually the person designated as the head of discipline or the head of nursing within the approved program of study [3, 4]. The term ‘academic nurse leader' is used to refer to other senior manager positions within a nursing department who have a leadership role but are not the disciplinary head.

Leading nurse education and research in the HE sector has become increasingly challenging over recent years with many universities in Australia and New Zealand having undergone academic organisational restructure to increase efficiency and reduce operational costs. These changes have resulted in many former standalone nursing faculties or schools becoming subsumed into larger entities or colleges, often lead by a non-nurse executive dean with a strong focus on corporate university outcomes [5]. COVID-19-induced cost saving measures in HE compounded the need for prudent fiscal restraint measures and placed additional pressures on university staffing budgets [6]. Changes across HE will continue to evolve as discussed in the recent Australian Universities Accord report, which noted that stronger leadership, planning and coordination is needed across the sector than is possible under the current system arrangements [2]. Supporting the appointment of nurse disciplinary leads to advance the discipline of nursing in this challenging environment needs to be informed by contemporary, evidence-based, human resource practices to ensure its subsistence into the future [2].

This scoping review aimed to explore what is known currently about the qualities, behaviours and characteristics of capable, strategic nurse disciplinary leads in HE. For the purpose of this study, qualities are defined as ‘a set of attributes or requirements'; behaviours are ‘the actions or activities of an individual'; and characteristics ‘relate to peculiar distinguishing features' [7]. Collectively, key qualities, behaviours and characteristics that exemplify the essence and culture academic leads should emulate are often referred to as ‘core attributes' [8].

## 2. Background

There are significant challenges in HE to grow the next generation of nurse disciplinary leads, a senior faculty manager appointment. Following the move of nurse education in Australia to the HE sector in the second half of the 20^th^ century, academic appointments at universities were seen as an attractive career alternative for RNs seeking to move away from clinical practice and shift work. In Australia and New Zealand, this workforce movement has decreased over the last decade due to a range of factors including improved remuneration and flexible work arrangements for nurses remaining in clinical practice and the requirement for nurse academics to hold higher degree qualifications for career advancement in academia [9–11]. Worldwide, there has been an increasing reliance on a casualised academic workforce to provide a flexible staffing establishment, manage fluctuations in student demand, as well as ensure that teaching staff have contemporary practice experience [2]. Streaming academics into teaching or research-only positions has further eroded the capacity for staff to gain a holistic experience of how disciplinary knowledge creation in HE informs curricula that traditional academic (teaching and research) roles afford. In addition, organisational restructures that have occurred in universities over the last decade have resulted in many nurse disciplinary leads no longer having budgetary and/or staffing oversight for teaching and research, thereby eroding the opportunity for heads of nursing departments to have full operational and decision-making scope across their faculty.

The HE sector also continues to be challenged by external influences including fallout from the COVID-19 global pandemic. In Australia and New Zealand, many universities have relied on the revenue generated from undergraduate nursing programs that have seen strong demand from both domestic and international students over many years. However, the impact of COVID-19 border restrictions led to a significant decrease in international student numbers in 2020 and 2021 [12], resulting in many universities offering early retirement schemes or forced redundancies, and nurse academics were among university faculty to leave the sector. Challenges also extended to postgraduate students, often the next generation of academic nurses, with relatively low stipends available to potential PhD candidates despite increasing costs of living as well as depleted funds available for professional staff development, mentoring and sabbaticals [2].

To strategically navigate the many complex challenges in HE and for the discipline of nursing to continue to thrive in the sector, capable, inspiring nurse disciplinary leads are needed to steer the discipline into the future. However, more needs to be known about the core attributes academics leading the discipline of nursing should demonstrate in order to significantly contribute to the growth, excellence and positive impact of nurse education, research and healthcare delivery.

## 3. Aim

This study by Slattery et al., the first phase of a large study [13], aimed to investigate what is known currently about the qualities, behaviours and characteristics of nurse disciplinary leads in HE leadership roles globally. Five research questions were identified (Table 1) to guide the exploration and synthesis of the current relevant international literature.

A scoping review method [14] was selected to address the research questions and explore an evidence-based set of core qualities, behaviours and characteristics that are exhibited by current nurse disciplinary leads and to determine what support structures and development opportunities emerging academic leaders require to become future nurse disciplinary leads. This method was chosen as it enabled the mapping of a wide-ranging set of the relevant literature and key concepts underpinning the research topic.

## 4. Method

This scoping review was reported using the preferred reporting items for systematic reviews and meta-analysis extension for scoping reviews (PRISMA-ScR) published in 2018 by Tricco et al. [15] and takes into consideration the recommendations of Bradbury-Jones and Aveyard [16] to ensure rigour whilst collating, summarising and reporting results within the fifth step of scoping review protocols. The scoping review protocol was published prospectively on the open science framework (reference: 10.17605/OSF.IO/QXAJN). Inclusion criteria consisted of articles that addressed one or more of the research questions and were published from January 2010 until December 2023 in English (primary language of the researchers). The inclusion period was chosen after careful consideration of the significant changes to HE organisational structures over the last 10 years to capture contemporary data aligned to the questions in Table 1. These changes include the financial impact of government funding models and market competition that has resulted in universities seeking senior leaders with contemporary leadership skills that can deliver sound education and research outcomes within budget targets. The search strategy was inclusive of the published and grey literature through searches within Google Scholar to minimise the effect of publication bias. Exclusion criteria consisted of articles not written in English due to the focus on nurse disciplinary leads in primarily English-speaking countries (e.g. Australia, New Zealand, the United Kingdom, and the United States of America) and those that focused on clinical nurse leadership rather than nurse disciplinary leadership in the HE sector.

During the early identification phase, the lead researcher conducted searches of the grey literature and databases including Medline, Embase, Emcare, Scopus and CINAHL using a combination of search terms that is Nurse; Leadership; Higher Education; Qualities; Behaviours; Characteristics; Education; Development; and Mentorship. This approach generated more than 10,000 articles; however, they primarily addressed leadership in clinical settings. Subsequently, the lead researcher amended the search strategy to focus on nurse disciplinary leads and HE. The lead researcher noted that the search output from Scopus and CINAHL was duplicated with the output of Embase. Embase was noted to hold the most comprehensive output from the search terms. Once excluding the duplications, it was noted that there were only clinical leadership articles remaining from the Scopus and CINAHL searches, and these were not relevant to this protocol. Scopus and CINAHL were subsequently excluded from the search strategy. Revised searches were undertaken in Medline, Embase, Emcare, ProQuest Dissertations and Theses Global and Open Access Theses and Dissertations (OATD) utilising broader search terms (Table A1) to capture workforce development and role-specific terminology, including Nurse; Leadership: Higher Education; Qualities; Behaviours; Characteristics; Tertiary; Discipline; Leader; Education; Mentor; and Staff Develop and associated Boolean phrases and combinations of these terms. The searches conducted in ProQuest Open Access Dissertations and Theses and OATD revealed no relevant papers. These searches continued to show a trend of clinical leadership articles, and subsequently, a final round of searches was conducted in Medline, Embase and Emcare focussing on the refined search terms listed in Table 2.

The screening phase focused on published primary studies, secondary studies and discussion articles from peer review journals which addressed one or more of the research questions. The searches conducted (as per Table 2) were further supplemented through the review of reference lists of included studies. Searches of Google utilising the terms in Table A1 determined that there was no grey literature to support the research questions.

**Figure 1:**
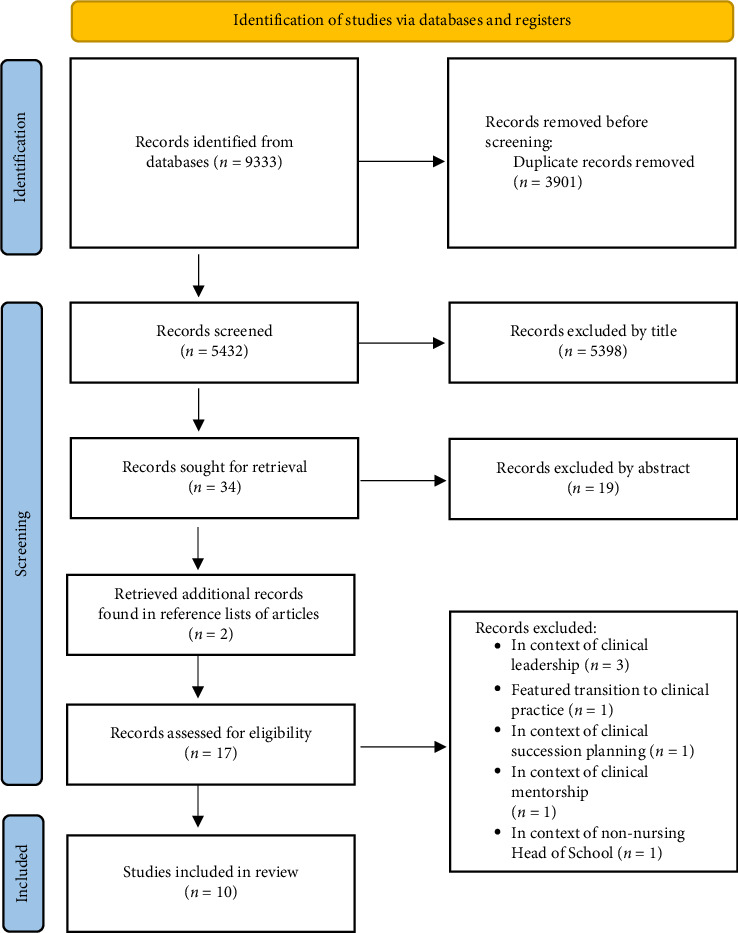
Scoping review process (adapted from Page et al. [17]).

The process utilised is detailed in Figure 1 and was informed by the work of Page et al. [17]. The search process resulted in a total of 5432 articles being identified. The lead researcher screened all articles by the title in EndNote. When reviewing the 5432 articles by the title, any article with a potentially ambiguous or unclear title was put forward for consideration by abstract to ensure that no relevant articles were missed. 34 articles were sought for retrieval and uploaded to Covidence. Two of the researchers (MS and CG) independently reviewed the 34 articles by abstract, with conflicts reviewed by the third researcher (RV). A strong theme of clinical leadership in hospital settings continued to be evident in the articles despite the refinement of the research terms. Fifteen articles were identified for in depth full-text review, and a further two articles were detected through a manual review of the reference lists to identify other literature that met the criteria. A total of 17 articles were then reviewed for full-text eligibility using Covidence and a worksheet the lead researcher created to capture further detail regarding the reasoning for each decision. The worksheet listed the inclusion and exclusion criteria and research questions (see Table A2). During this process, six articles were excluded because they did not address the questions listed in Table 1 and had a clinical workplace focus, as opposed to a focus on the HE sector. One further article was excluded after review by the third researcher (RV) due to the context of the article focussing on a non-nursing Head of School/Program Director. As a result, a total of 10 articles were identified for inclusion in this review as depicted in Figure 1.

A data extraction tool (Table A3) was created in Microsoft Word to identify and analyse how each article addressed one or more of the research questions, and a content analysis was conducted, informed by the work of Grbich [18]. The researcher's comments regarding key findings from the full-text review (as mapped to the research questions) were populated within the table. An adapted data richness scale derived from the work of Ames et al. [19] was utilised to assess data richness (detailed in Table 3) of all 10 articles, where a score of one indicated that very little data were represented and a score of five indicated that a large volume and depth of data were represented [19]. The adapted scale was utilised to provide in-depth insights into the data. Data extraction was performed using the table specified in Table A3, detailing the data richness score and specific mapping to the scoping review questions.

## 5. Findings

The study identified 10 articles that addressed one or more of the research questions. All articles originated from the United States of America, England or Australia. The articles included qualitative studies, interpretative phenomenological studies and a sequential mixed method study. The study size ranged from 10 to 58 participants. All articles had a score of between 2 and four on the data richness scale (Table 3). The findings of the study are presented under each research question.

### 5.1. What Are the Qualities, Behaviours and Characteristics Required for a Nurse Disciplinary Lead?

All articles mentioned some aspect of core attributes required by nurse disciplinary leads (Table A4). However, there was a lack of clarity and definition of terms used, no refinement to categorise like-terminology, and limited consideration of how the terms were captured within qualitative data sets. Importantly, Wilkes et al. [20] highlighted that there is a plethora of terms used for various attributes, and these qualities, behaviours and characteristics could be broadly categorised as positional or personal characteristics. Wilkes et al. [20] determined that both categories were needed due to the complexity of the nurse disciplinary lead role and identified positional leadership characteristics including the ability to be strategic, operational and manage academic and human resources. Behavioural, interpersonal and relational characteristics were also identified; however, there was confusion around definitions and categorisation of qualities, behaviours and characteristics due to the abundance of terms used [20–26]. Specific core attributes identified in the literature included efficient time management, the ability to reflect, perseverance, ability to weigh costs and benefits, learning the context of the HE environment, cultivating relationships, getting involved with others, preserving authenticity, creating environments for change, coping with being thrust into leadership, enjoying creative freedom, experiencing professional growth, a sense of ‘calling' and the ability to relate to others in a new way [10, 21, 24, 26–28]. Pearsall et al. [24] and Young et al. [26] identified the contrasting skills of minimising risk taking while knowing when to take risks.

### 5.2. What Are the Qualifications Required to Work as a Nurse Disciplinary Lead?

Three articles discussed qualifications required for nurses working in HE [10, 21, 25]. To be a nurse disciplinary lead in HE, universities have traditionally required a doctoral degree, and authors, including Delgado and Mitchell [21], identified requisite postgraduate qualifications for nurses working in HE as a barrier for career advancement. Nurses are more likely to enter HE later in their career, and the literature also highlighted the clinical focus of qualifications that many nurses enter HE with, as opposed to postgraduate degrees with an education and/or a research focus required for advancement in the university sector. Bouws et al. [10] argued that the low uptake of the PhD study by nurses constrained their recruitment into academic leadership roles. Ross et al. [25] highlighted the importance of doctoral prepared nurses to assume nurse disciplinary lead roles for the advancement of the practice-based discipline in HE, and Delgado and Mitchell [21] identified that nurses working in HE felt disadvantaged by not holding the ‘right' degree. Ross et al. [25] argued that nurse disciplinary leads must develop and promote a narrative which demonstrates the ability of academic nursing to influence quality of care.

### 5.3. What Are the Intrinsic and Extrinsic Factors Which May Affect an Individuals' Ability to Succeed as a Nurse Disciplinary Lead?

There were eight articles which identified a range of intrinsic factors which may affect an individuals' ability to succeed as an academic nurse leader in HE [10, 21, 22, 24–28]. These included having a specific leadership skillset that facilitated a smooth transition to the nurse disciplinary lead role, such as an ability to lead change, professional values, interprofessional skills and self-awareness [10]. Extrinsic factors identified that may affect an individuals' ability to succeed as a nurse disciplinary lead included the impact of experienced clinical nurses commencing HE roles later in their careers restricting their opportunities for career progression in traditional HE hierarchical structures [22]. As nursing is a female-dominated workforce, challenges faced by women in the workplace, including child and family carer roles, were also perceived as inhibiting career progression [22, 26].

### 5.4. Does Mentorship Have a Positive Impact on Academic Nurse Leadership Skill Development?

A theme of mentorship and its highly positive impact on academic nurse leadership skill development was identified in six articles [10, 21, 22, 24, 26, 28]. The articles identified that sharing of personal stories and helping the mentee to develop their professional identity would positively impact individuals [21, 26, 28]. Delgado and Mitchell [21] suggested that mentorship was the most beneficial driver to support leadership development for emerging nurse disciplinary leads. There was strong emphasis on the recommendation that mentors be assigned to ensure that the chosen mentor was knowledgeable about leadership development and best practices [21, 26, 28]. Further activities identified to support the development of nurse disciplinary leads in HE included undertaking formal professional leadership programs and provision of funding for scholarships specific to leadership training and development [24]. Pearsall et al. [24] found that academic and clinical nurse leaders sharing strategies to address leadership concerns helped to support nurse leader transition.

### 5.5. What Type of Framework Could Assist Supporting Nurses Into the Role of a Nurse Disciplinary Lead?

In only one article, Wilkes et al. [20] addressed whether a framework approach could assist nurses in HE to develop the qualities, behaviours and characteristics required for disciplinary leadership in HE. They argued that the leadership attributes of successful nurse disciplinary leads could be classified as either positional or personal and that this awareness enables the creation of structured programs which are essential to developing and understanding these attributes in future nurse disciplinary leads [20].

## 6. Discussion

This scoping review identified that a scarcity of the evidence-based literature exists to address the qualities, behaviours and characteristics needed by nurse academics in the HE sector. Aside from mentorship, there is limited literature on the support structures and development opportunities emerging leaders require to become future nurse disciplinary leads. Internationally, the majority of the literature on leadership development for nurses pertains to clinical leadership, and while key leadership qualities can be transferable, the context of how universities operate requires specific knowledge of the HE sector, requisite qualifications and experience in HE education and research to ensure that nursing as a discipline continues to thrive.

The current literature on this topic indicates that extrinsic factors, including the nature of nursing work as an applied profession, nurses coming into HE careers at a later age, challenges faced by women in the workplace, including child and family carer roles, and the requirement of requisite qualifications needed for career advancement in HE, all present barriers and challenges for emerging nurse disciplinary leads in this sector [22]. Whilst the development of a framework may support the establishment of benchmarks for nurse disciplinary leadership roles, such a framework would also require clear definitions and consideration of personal qualities, behaviours and characteristics essential for the individual to be successful.

The Australian Universities Accord Report [2] highlighted that investment in staff at all stages of their careers is essential to foster the future leaders of Australia's universities, TAFEs and VET providers. Nursing needs outstanding nurse disciplinary leads in HE to advance the discipline and ensure that there is strong correlation between the research, education and practice nexus. Further research is required to identify the qualities, behaviours and characteristics (core attributes) required by future nurse disciplinary leads that could underpin professional development programs. This research could include the establishment of a consensus view on a core set of qualities, behaviours and characteristics or indeed a codesigned set of metrics that future nurse disciplinary leads will require and the development of an evidence-based framework that articulates the support structures. The support structures could include targeted education or leadership programs that emerging nurse leaders in HE can undertake to enhance and enable leadership success through accredited and nonaccredited education pathways. These programs could be designed to align with recommendation 31 from the Australian Universities Accord Report [2]. Findings from further research could also contribute to new policy directions for key bodies such as the Council of Deans of Nursing and Midwifery (Australia & New Zealand) that supports recruitment strategies, succession planning and mentoring programs and by identifying and targeting emerging nurse leaders who demonstrate the core attributes which facilitate nurse disciplinary lead success. Seven of the articles identified in this review were from the United States of America. Further research could be undertaken to examine the cultural basis for these findings and transferability to other HE institutions in Australia, New Zealand and the United Kingdom.

All articles had a strong focus on qualitative data. Further empirical work, including prospective quantitative studies, are needed to determine the impact of the core attributes on staff retention, satisfaction and development. Correlational or quasi-experimental research could examine the implementation of mentorship programs or professional development to enhance the core attributes of future nurse disciplinary leads.

### 6.1. Strengths and Limitations

This scoping review used precise and transparent methods throughout the review process and aligned to the PRISMA-ScR [15]. To ensure a broad search of the literature, the search strategy included five electronic bibliographic databases, and search terms were amended to ensure precision in identifying the literature that met the inclusion criteria. The selection of studies was guided by a protocol reviewed by the research team with expertise in conducting scoping reviews and prospectively published on the Open Science Framework. The data extraction tool was used to mitigate any potential reviewer bias [14] when characterising and interpretating the included articles.

While the researchers made every endeavour to ensure the rigour of the review design, there were limitations. The review included articles published in English only due to the focus on nurse disciplinary leads in primarily English-speaking countries (e.g. Australia, New Zealand, the United Kingdom, and the United States of America), and there may have been relevant articles published in other languages that were missed. The researchers involved in this study are solely English speakers, and due to resource constraints (including the lack of funding and multilingual expertise) and to avoid the potential for language bias, only articles written in English were included. Further research could explore parallels to other continents with similar HE nurse leadership roles. Throughout the initial searches, it became evident that there was a strong theme of clinical leadership which did not address the key research questions. A focus on clinical leadership was set as a parameter for exclusion and was considered when the articles were reviewed by the title.

Further limitations included the confusion around definitions and categorisation of qualities, behaviours and characteristics. For example, Delgado and Mitchell [21] highlighted intelligence as a desirable quality; however, the article does not specify whether this relates to emotional, cognitive or spiritual intelligence [21].

## 7. Conclusion

This scoping review aimed to explore what is known currently about the qualities, behaviours and characteristics of nurse disciplinary leads in HE. Ten articles met the inclusion criteria, and on analyses of the literature, this review found that a range of intrinsic and extrinsic factors exist that limit the opportunity for the next generation of outstanding nurse disciplinary leads to emerge. Further research is needed to identify an agreed set of core attributes to identify and support the professional development and readiness of future generations of nurse disciplinary leads. By identifying the qualities, behaviours and characteristics which underpin the success of nurse disciplinary leads, we are able to identify opportunities to support the growth and readiness of future generations of nurse disciplinary leads and challenge the current deterrents to HE nurse academic career pathways.

## Figures and Tables

**Table 1:** Scoping review questions.

1. What are the qualities, behaviours and characteristics required for a nurse disciplinary lead?
2. What are the qualifications required to work as a nurse disciplinary lead?
3. What are the intrinsic and extrinsic factors which may affect an individuals' ability to succeed as a nurse disciplinary lead?
4. Does mentorship have a positive impact on academic nurse leadership skill development?
5. What type of framework could assist supporting nurses into the role of a nurse disciplinary lead?

**Table 2:** Summary of search terms.

Leadership/
((Nurse^∗^ or midwif^∗^) adj3 (leader^∗^ or co?ordinat^∗^ or manager^∗^ or supervis^∗^)).mp. [mp = title, abstract, original title, name of substance word, subject heading word, floating subheading word, keyword heading word, organism supplementary concept word, protocol supplementary concept word, unique identifier, synonyms]
Education/and nursing/and nursing education/
Academ^∗^.mp
Faculty.mp or university/
Mentorship/

**Table 3:** Data richness scale (adapted from Ames et al. [19]).

Score	Measure
1	Very little qualitative data presented that relate to the synthesis objective. Those findings that are presented are fairly descriptive
2	Some qualitative data presented that relate to the synthesis objective
3	A reasonable amount of qualitative data that relate to the synthesis objective
4	A good amount and depth of qualitative data that relate to the synthesis objective
5	A large amount and depth of qualitative data that relate in depth to the synthesis objective

**Table 4:** Detailed search terms.

#	Searches
1	Exp nurse/
2	Limit 1 to (English language and yr = “2010-Current”)
3	Leadership/
4	Limit 3 to (English language and yr = “2010-Current”)
5	Higher education/
6	Limit 5 to (English language and yr = “2010-Current”)
7	Qualities/
8	Limit 7 to (English language and yr = “2010-Current”)
9	Behaviours/
10	Limit 9 to (English language and yr = “2010-Current”)
11	Characteristics/
12	Limit 11 to (English language and yr = “2010-Current”)
13	2 and 4 and 6
14	8 and 10 and 12
15	Tertiary/
16	Limit 15 to (English language and yr = “2010-Current”)
17	((Nurse^∗^ or midwfi^∗^) adj3 (leader^∗^ or co?ordinat^∗^ or manager^∗^ or supervis^∗^)).mp. [mp = title, abstract, original title, name of substance word, subject heading word, floating subheading word, keyword heading word, organism supplementary concept word, protocol supplementary concept word, rare disease supplementary concept word, unique identifier, synonyms]
18	Limit 17 to (English language and yr = “2010-Current”)
19	6 and 18
20	4 and 18
21	Discipline/
22	Limit 21 to (English language and yr = “2010-Current”)
23	2 and 22
24	(Educat^∗^ or curriculum or teach^∗^ or learn^∗^ or train^∗^ or mentor^∗^ or staff develop^∗^ or workforce develop^∗^ or develop^∗^ staff or develop^∗^ workforce or professional^∗^ develop^∗^ or succession plan^∗^).mp. [mp = title, abstract, original title, name of substance word, subject heading word, floating subheading word, keyword heading word, organism supplementary concept word, protocol supplementary concept word, rare disease supplementary concept word, unique identifier, synonyms]
25	Limit 24 to (English language and yr = “2010-Current”)
26	22 and 25

**Table 5:** Scoping review checklist (full-text review).

Specific questions to be pursued during the scoping review	Yes	No	Comments
What are the qualities, behaviours and characteristics required for a disciplinary lead?			
What are the qualifications required to work as a disciplinary lead?			
What are the intrinsic and extrinsic factors which may affect an individuals' ability to succeed as an academic nurse leader?			
Does mentorship have a positive impact on academic nurse leadership skill development?			
What type of framework could assist supporting nurses into the role of an academic nurse leader?			

*Inclusion criteria*			
Published from January 2010 until current date in English.			
Primary studies/published research studies.			
Secondary literature reviews.			
Grey literature (such as role descriptions)			
Australian and New Zealand university websites, SEEK website, and core attributes of academic staff.			

*Exclusion criteria*			
Written in a language other than English			
Focus point on clinical leadership			

**Table 6:** Scoping review findings.

Author/Year	Country	Method	Study participants	Findings	Data richness scale [19] (where 1 is lowest, and 5 is highest)	Mapping against questions in Table 1.0
[10]	The United States of America	Grounded theory and face-to-face and phone interviews	18 interviews with nurse deans, directors and chairpersons	Q1: Indicates that the characteristics of the academic leader's personal journey are important to understand in order to appreciate what may be required to successfully transition future leaders and retain them in their roles. Q2: Discusses Masters degrees being clinically focused—low uptake of doctorally prepared nurses to take on the role of academic leaders. Q3: Role fulfilment effected by intrinsic factors—having a skillset and sense of calling. Extrinsic factors—experiencing a variety of relationships, seeing positive change, enjoying creative freedom, experiencing professional growth and having the support from administration. Q4: Discusses the value of the American Association of colleges of nursing mentorship programs plus support from faculty and upper administration.	3	Q1, Q2, Q3 and Q4

[21]	The United States of America	One-time, cross-sectional, online survey	52 faculty members from 12 universities	Q1: Integrity/fairness, intelligence and vision. Q2: Mentioned leadership programs—but not an uptake from participants. Discusses the doctor of nursing practice. Highlights that some respondents felt disadvantaged that they did not have the ‘right degree' [21], p12. Q3: Barriers to obtaining a leadership position examined from two perspectives. Participants answers, while not thematically analysed, fell into easily distinguished categories: Time management and support, which had three subcategories (administrative, collegial and mentors). Personal barriers also discussed. Q4: Describes mentoring as the most helpful leadership experience (53.5%).	3	Q1, Q2, Q3 and Q4

[22]	Australia	Sequential mixed-methods, online surveys and semistructured interviews	23 early career nursing academics	Q1: Participants were able to articulate the qualities of effective leadership, and these included showing respect and being a respectful person. Q3: The paper highlights some of the issues and challenges associated with the ageing workforce in relation to transition into a new work context and in developing leadership capacity in academia. Also highlights some of the challenges faced by women. Q4: In the context that while leadership programmes targeted at developing and mentoring the upcoming early career nursing leaders in the clinical setting, these are not subsidized for those working in an academic setting.	2	Q1, Q3 and Q4

[27]	The United States of America	Unstructured audio-taped interviews	23 nurse faculty leaders	Q1: Reflecting, persevering through difficulties, learning to relate to others in new ways, facing challenges, making difficult decisions, looking inward, reflecting and persevering through difficulties. Q3: To some extent in terms of the challenges one needs to overcome, reflecting and persevering through difficulties and learning to relate to others in a new way.	2	Q1 and Q3

[23]	The United States of America	Descriptive, correlational mixed methods and online survey	58 people who worked full-time, part-time or adjunct in a nursing faculty position	Q1: Participants described desirable behaviours (including open, honest and transparent communication) and ineffective leadership behaviours (including the lack of trust leading to micromanagement, abusive supervision and authoritarian leadership).	2	Q1

[24]	The United States of America	Interpretive phenomenological study and telephone interview	24 nurse faculty	Q1: This study was a follow-up study from the United States group with Young et al. To further explore the themes from that study—this one looked at the specific phenomenon of risk-taking in leadership; 7 of the original 11 members of the research team reconvened for this follow-up study. Q3: Intrinsic factors identified were ‘doing their homework' to minimize their risk taking: Weighing the costs and benefits, learning the context and cultivating relationships. Q4: Yes, but as a recommendation ‘this can be accomplished through developing formal programs of leadership development for faculty in various stages of their careers, establishing mentorship experiences, funding scholarship related to leadership development in academia and seeking opportunities to build leadership initiatives that bring nurse leaders from practice and academia together to collaboratively share strategies to address their leadership challenges' [24], p32.	3	Q1, Q3 and Q4

[25]	England	Qualitative design and open ended telephone interviews	10 academic nurse leaders	Q1: Qualities embracing emotional intelligence, interpersonal skills, courage and tenacity identified as important for nursing academic leaders. ‘Standing up and speaking out', resilience, pioneering and breaking new ground [25], p1344. Q2: Suggests the need for doctoral prepared nurses to take on the role of the dean. Q3: Explored the scope of the role and the personal, professional and academic challenges for nurses in leadership roles in universities in the United Kingdom.	3	Q1, Q2 and Q3

[28]	The United States of America	Interpretative phenomenological study	23 nurse faculty leaders	Q1: Being involved with others, advancing reform, serving as a symbol and creating an environment for change. Q3: Suggest that the common experience of participants advancing reform illuminates how nurse educators become leaders by getting involved with others, struggling to serve as a symbol and preserve authenticity and creating environments for change. Q4: Indicates that the assignment of mentors is recommended.	2	Q1, Q3 and Q4

[20]	Australia	Qualitative design and semistructured interviews	30 nursing deans	Q1: Indicates that the literature outlines the characteristics of passion, vision, courage, integrity, credibility, adaptability, perseverance, creativity, good communication skills, open mindedness, inspiration and commitment. Leadership attributes of a successful nursing dean nominated by participants and categorised as positional or personal. Delegation, working in and for an organisation, teamwork, collaborating with internal and external stakeholders and organisational and staff management. Q5: Outlines leadership attributes of a successful nursing dean—categorised as positional or personal.	4	Q1 and Q5

[26]	The United States of America	Interpretative phenomenological study	21 nurse faculty leaders	Q1: Being thrust into leadership, taking risks, facing challenges, reflecting, persevering, learning to relate with others in new ways, advancing reform and being in service to a larger cause. Q3: Being thrust into leadership, taking risks and learning to face challenges are vital experiences; formal preparation that supports and encourages the development of nurse faculty leaders. Q4: Indicates that the assignment of mentors is recommended.	2	Q1, Q3 and Q4

**Table 7:** Core attributes.

Date of publication	Authors	Country	Core attributes
2022	Hudgins, T., Brown, K., Layne, D., and Stephens, T.	The United States of America	Modelling the way, inspiring a shared vision, challenging the process, enabling others to act and encouraging the heart Open honest communication - Openness and honest communication - Openness - Being open and direct on expectations - Increased communication - Transparency - Being open and honest about changes that are occurring Accountability - Increased accountability by leadership of faculty and staff - Holding true to the policy regarding student behaviour - Availability - Leadership taking responsibility for addressing personnel issues - Responsibility to address issues - Hold other accountable to their duties and not ignore faculty that are not following the rules Demonstrates professional behaviours and competence - Trust - Ethical behaviours - Intelligence - Respectful - Professionalism - Honesty, consistency, civility and academic integrity - Less favouritism toward certain faculty Promotes teamwork and collaboration - Promoting teamwork - Agenda more holistically focused on the interests of all coworkers - Collaboration Anticipates and plans ahead - Improved planning—less spur of the moment - Anticipate needs Promotes collaboration - Allowing all faculty to have a voice in processes, procedures, and opportunities - Leadership embracing perspectives outside of the institution Advocates for faculty and department - Advocacy for needed resources for the department Orientation, mentorship and staff development - Orientation and training for new faculty - Having a mentorship program for new faculty - Providing educational updates and resources - Promoting scholarship for faculty Guidelines, policies and expectations - Clear guidelines and policies - Clear job expectations - Understand the workload Faculty trust - Freedom to follow policies and guidelines without being undermined by management and lack of support for faculty decisions - Trusted me to do the job on my own Respect and meaningful recognition - Praise for adjuncts - More recognition and gratitude - Equal opportunity and recognition - Sincere inquiry about availability when establishing meetings, etc. Diversity and inclusion - To stop division and stay diverse Recognising nursing and our importance to the university
2020	Bouws, M., Madeira, A., and Streberger, A.	The United States of America	Personality traits, having a skillset, a sense of calling, extrinsic factors of fulfilment, enjoying professional growth, reaching out to others for assistance in adapting to their role as an academic nurse leader, having support from upper administration, collaborating with other professionals and mentoring others
2016	Delgado, C., and Mitchell, M.	The United States of America	Integrity, communication clarity, problem-solving ability, research track record, experience gaining grants, friendliness, time management, change leadership, management skills, ability to make interpersonal connections, empathy, understanding of the administrative structure, ability to build partnerships, mentoring skill, intelligence, strategic thinking, faculty support, motivational ability, fairness and vision
2016	Halcomb, E., Jackson, D., Daly, J., Gray, J., Salamonson, Y., Andrew, S., and Peters, K.	Australia	Articulate a clear view of leadership
2015	Wilkes, L., Cross, W., Jackson, D., and Daly, J.	Australia	Faculty development, team building, conflict resolution, financial planning, delegating, implementing change, decision-making, flexible, consistent, equitable, nurturing, resourceful and courageous Committed, communicator, consistent, courage, credibility, equitable, excite, facilitator, fair, flexible, innovative, inspire, look for opportunities, not always popular, not infallible, nurture, passionate, patience, push boundaries, reflective, resilient, resourceful, role model, self-knowledge, share, support, transparent, value relationships, vision, change agent, conflict resolver, communicator, decision maker, financial planner, good manager, delegator, role model, team builder, vision, pushes boundaries, focus on staff and students, faculty developer, connect to external stakeholders, credibility, promoter/advocate of nursing and be a nurse
2014	Pearsall, C., Pardue, K., Horton-Deutsch, S., Young, P., Halstead, J., Nelson, K., Morales, M.L., and Zungolo, E.	The United States of America	Taking well-calculated risks, including acting to make unpopular decisions, doing the right thing, innovating and moving one's career forward
2013	Ross, F., Marks-Maran, D., and Tye, C.	England	Skill of navigation to weave a path around both sets of agendas, leading from the front and pushing from the rear
2011	Stiles, K., Pardue, K., Young, P., and Morales, M.L.	The United States of America	Being involved with others, advancing reform, serving as a symbol and creating an environment for change
2011	Young, P., Pearsall, C., Stiles, K., Nelson, K., and Horton-Deutsch, S.	The United States of America	Being thrust into leadership, taking risks, facing challenges, reflecting, persevering, learning to relate with others in new ways, advancing reform and being in service to a larger cause
2010	Horton-Deutsch, S., Young, P., and Nelson, K.	The United States of America	Reflecting, persevering through difficulties, learning to relate to others in new ways, facing challenges, making difficult decisions, looking inward, reflecting and persevering through difficulties

## Data Availability

Data sharing is not applicable to this article as no datasets were generated or analysed during the current study.

## Appendix A

The variation in the level of detail within the below table is a reflection of the depth of information provided in the original articles. Some articles offered more comprehensive insights, while others focused on broader or more concise points. Each article was summarised in line with the content that was available.
